# Combination of Advanced Platelet-Rich Fibrin and Pentoxifylline/Tocopherol as a Novel Preventive Option in Osteoradionecrosis: A Case Report

**DOI:** 10.1055/s-0042-1750777

**Published:** 2022-10-04

**Authors:** Pasinee Vorakulpipat, Suphachai Suphangul, Pornpoj Fuangtharnthip, Shahram Ghanaati, Chakorn Vorakulpipat

**Affiliations:** 1 Department of Advanced General Dentistry, Faculty of Dentistry, Mahidol University, Bangkok, Thailand; 2 Department of Oral and Maxillofacial Surgery, Faculty of Dentistry, Mahidol University, Bangkok, Thailand; 3 Department for Oral, Cranio-Maxillofacial and Facial Plastic Surgery, Johann Wolfgang Goethe University, Frankfurt, Germany; 4 FORM-Lab, Frankfurt Orofacial Regenerative Medicine, Department for Oral, Cranio-Maxillofacial and Facial Plastic Surgery, Johann Wolfgang Goethe University, Frankfurt, Germany

**Keywords:** osteoradionecrosis, bone defect, bone regeneration, platelet-rich fibrin, pentoxifylline, tocopherol

## Abstract

Osteoradionecrosis (ORN) of the jaws is an uncommon complication of radiation therapy that seriously affects the oral and maxillofacial region. Management of ORN is intrinsically difficult and treatment effects are unpredictable. ORN can be treated with pentoxifylline/tocopherol and autologous platelet concentrates to promote wound healing. Furthermore, the low speed of relative centrifugal forces platelet-rich fibrin (PRF + ) has been shown high efficacy for ORN. A 72-year-old male patient with history of radiation treatment for squamous cell carcinoma in the left side of the tongue. Six years after the treatment, his upper right first molar tooth (no. 16) was surgically extracted due to persistent pain. A few months following the extraction, intraoral examination showed gingival inflammation, and pain when palpation around the edentulous area of tooth no. 16. Radiological examination revealed retained root of 16 with radiolucent area and horizontal bone loss around upper right second molar tooth (no. 17). Pentoxifylline and tocopherol were given for a week before the surgical operation and were continued for 8 weeks after the operation. Retained roots of teeth no. 16 and 17 were removed and the sockets were debrided, the advanced PRF+ (A-PRF + ) membranes were placed followed by primary wound closure. Following 2 weeks of treatment, the mucosa healed and progressed to complete mucosal coverage at 2 months with no pathological findings or ORN progression. At 6-month follow-up, clinical and cone-beam computed tomography (CBCT) revealed no pathology. Our case demonstrates that the combination of pentoxifylline/tocopherol and the A-PRF+ surgical approach can be useful for wound healing and prevention of ORN.

## Introduction

According to the Global Burden of Cancer Study, a quarter million people have suffered from oral cancers worldwide. 1 Radiotherapy is recommended for the definitive or palliative treatment of oral cancer patients alone or in conjunction with surgery. However, radiotherapy has considerable drawbacks as it results in acute and late side effects. Acute side effects, such as moist desquamation, skin erythema, taste loss, and mucositis are often weakening but resolve with time. However, the late side effects, such as radiation caries, trismus, xerostomia, myelitis, fibrosis of the skin, and osteoradionecrosis (ORN) are more significant and be life-long problems for the cancer survivor patients. 2

ORN of the jaws defined as the exposed irradiated bone that fails to heal over a period of 3 months without any evidence of persisting or recurrent tumor. The prevalence of ORN has been reported to be 5 to 15% and commonly affects patients over 55 years of age. 3 The most common pathophysiologic models of ORN are described by hypoxic-hypocellular-hypovascular theory and radiation-induced fibroatrophic (RIF) theory. These debilitating conditions can arise on their own but they are frequently triggered by soft and hard tissue manipulation, such as tooth extraction and trauma.

The management of “at-risk” individuals for ORN is still a challenge for the physicians; prevention and education are essential. The dental treatments can be performed but precautions need to be taken when performing the oral surgical procedures. There has been no clear consensus on the prevention of ORN but primary closure of wounds, systemic antibiotics, and antiseptic mouthwashes are important considerations. Novel medications to prevent the ORN are promising, as hyperbaric oxygen (HBO) therapy is no longer recommended. 4 In addition, HBO therapy requires special equipment and long course of treatment, limiting the accessibility.

Combination of pentoxifylline and tocopherol has been introduced as a noninvasive option for the ORN prevention. Pentoxifylline is an inhibitor of tumor necrosis factor-α (TNF-α). It improves the erythrocyte flexibility, causes blood vessels dilatation, inhibits inflammatory reactions, decreases the proliferation of human dermal fibroblasts, helps extracellular matrix synthesis, and boosts collagenase activity. Tocopherol (vitamin E) is used to scavenge the reactive oxygen species produced by preserving the cell membranes against peroxidation of lipids, causes partial suppression of transforming growth factor (TGF)-1 and helps in developing the genes procollagen that decrease fibrosis under oxygen stress. 5 These two drugs act synergistically as powerful antifibrotic agents, causing a 66% regression of the surface area of RIF process following 12 months of therapy. 6

In addition, platelet-rich fibrin (PRF), originally developed by Choukroun et al 7 in 2001, was introduced as the blood concentrate system that did not require the use of extra anticoagulants. PRF-based matrices contain a variety of inflammatory cells, such as leukocytes and platelets, as well as plasma proteins embedded in a fibrin network. 8 The components of PRF-based matrices are recognized to have an essential role in the wound healing process. Local PRF administration can minimize the postoperative discomfort and complications. Advanced-PRF+ (A-PRF + ) is a new PRF-based matrix developed by Ghanaati et al. 9 in 2014. The low speed of relative centrifugation force demonstrated enhanced growth factor release within PRF-based matrices. Literature suggests that PRF is useful in poor healing potential, such as in ORN and its properties, they are useful as an adjunct in the prevention and treatment of ORN. 10 11

In this case report, we aim to present the preventive strategy and outcomes of A-PRF+ as a surgical adjunct for ORN.

## Case Report

A 72-year-old man presented to an advanced dentistry department of an academic tertiary hospital in Thailand, with persistent pain at edentulous area of the upper right first molar. The patient was previously diagnosed with squamous cell carcinoma in the left side of the tongue with minimal submucosal invasion (staging T2N0M0) 6 years prior, treated with wide excision with modified radical neck dissection (mandibular split). External beam radiation therapy was performed at a total dose of 5,940 cGy in 33 Days.

In the following 6 years after radiotherapy, he has had severe hyposalivation and multiple dental caries. The tooth no. 16 was surgically extracted due to caries and persistent pain. A few months following extraction, he presented to our clinic with trouble chewing meals and expressed a desire for a new denture. An intraoral examination showed gingival inflammation and tenderness with palpation around the edentulous area of tooth no. 16. Upper right second premolar tooth (no. 15) had no symptoms. Upper right second molar tooth (no. 17) was unoccluded tooth with supraeruption and first degree mobility. The furcation involvement grade I at buccal aspect, with horizontal bone loss at apical one-third of root length and root caries, was detected.

**Fig. 1:**
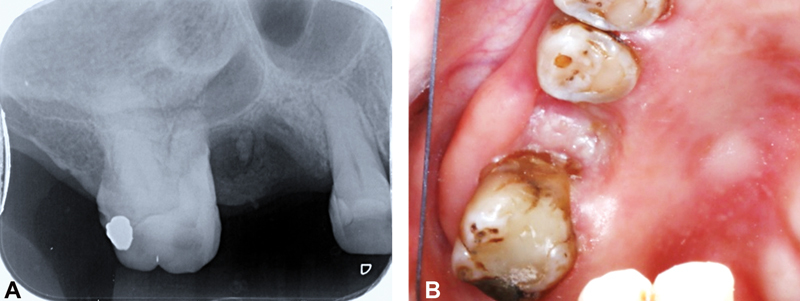
( **A** ) Preoperative periapical radiographic examination showed retained root of 16 with radiolucent area; ( **B** ) clinical findings showed unhealed edentulous area.

Radiological examination revealed retained root of no. 16 with radiolucent area and horizontal bone loss around tooth no. 17. Our prosthodontist recommended that the tooth (no. 17) had to be removed because of poor prognosis with no space for opposing denture. Retained roots of teeth no. 16 and 17 were removed and the sockets were debrided, the A-PRF+ membranes were prepared and placed as described in the following sections followed by primary wound closure ( Fig. 1 ).

### Advanced-Platelet-Rich Fibrin+ Preparation

**Fig. 2:**
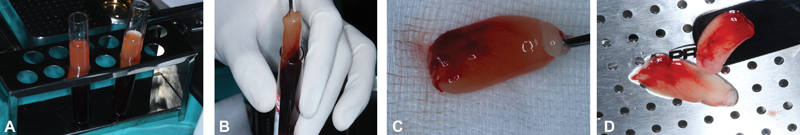
( **A** ) PRF were incubated for 5 minutes at room temperature; ( **B** ) the maturated PRF clot was removed from tube with a sterile tweezer; ( **C** ) the fibrin clot was separated from the red blood cell fragment, using a scissor; ( **D** ) the A-PRF+ membrane after incubating in PRF Box grid for 5 minute. A-PRF + , advanced platelet rich fibrin

A-PRF+ application was used to stimulate and accelerate wound healing as described by Ghanaati et al. 9 Briefly, 20-mL peripheral blood was drawn from the patient and collected in two 10-mL sterile glass tubes (A-PRF tubes process for PRF, Nice, France; Mectron, Cologne, Germany) without additional anticoagulants then placed in a centrifuge machine (Duo centrifuge, process for PRF, Nice, France; Mectron, Cologne, Germany). The centrifuge had a fixed angle rotor with a radius of 110 mm with no brake. The preparation stages were performed at room temperature using the following protocols: 1,300 rpm; 8 minutes; and 208 g. The centrifugation process completed automatically following the centrifugation time, and the centrifuge stops in 2 to 5 seconds. Clots were gently removed from the tubes and separated from the red blood cell fraction using sterile scissors after incubation for 5 minutes for clot development. The clot A-PRF was then placed on the PRF Box grid (Process for PRF, Nice, France) and covered with the lid ( Fig. 2 ).

### Surgical Procedure

Surgical removal of remaining root no. 16 and tooth of no. 17 were performed using aseptic techniques under local anesthesia (using Articaine 4% with Epinephrine 1:100,000 1.7 mL). Access to target site was achieved by raising a full-thickness mucoperiosteal flap to fully exposed bone area of no. 16. Then the retained root and tooth no. 17 were gently removed. 5 The granulation tissues were removed using a curette and the irrigation was performed using normal saline. Afterward, the periosteum was released to ensure a tension-free wound closure. The A-PRF+ membranes were then placed into the denuded bone, followed by primary wound closure using resorbable suture, Vicryl 4–0.

**Fig. 3:**
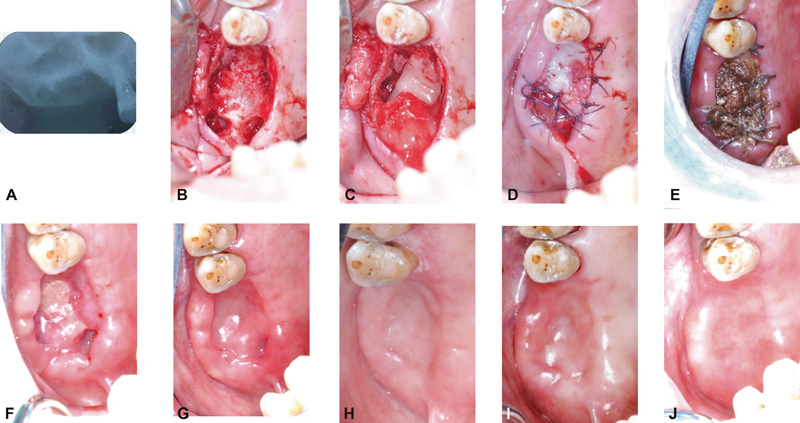
( **A** ) Immediate postoperative periapical radiographic examination showed that the residual root was completely removed. ( **B** ) A full thickness mucoperiosteal flap was performed and the tooth 17 has extracted; ( **C** ) A-PRF+ application on denuded bone; ( **D** ) Tension-free wound closure was achieved; ( **E–J** ) At 1, 2, 4, 8, 16, and 24 weeks of postoperative follow-up J caption is repeated. Please remove.

The patient was given the postoperative instructions and a course of antibiotics (Amoxycillin 500 mg, thrice a day) for 7 days. Because the patient has multiple risk factors of developing postextraction ORN, a pharmacologic approach was devised. To prevent the development of ORN, pentoxifylline of 800 mg and tocopherol 1,000 IU were given daily for a week before the surgical operation and were continued until the 8-week course was finished ( Fig. 3 ). 5 6

### Follow-up

**Fig. 4:**
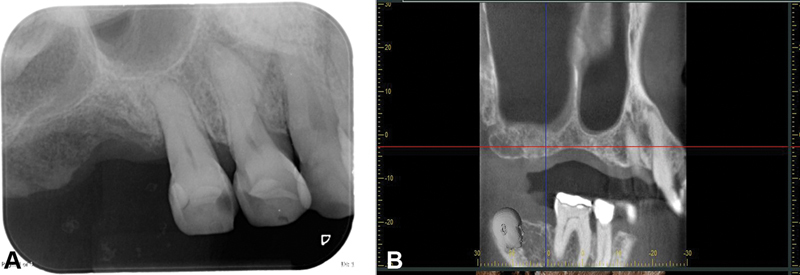
( **A** and **B** ) 6 months postoperative periapical radiographic and CBCT examination showed the new bone formation without pathological findings. CBCT, cone-beam computed tomography.

At regular follow-up visits, no reports of pain or edema were observed. At 2 weeks' visit, examination revealed early epithelization and progressed to complete mucosal coverage at 2 months. At 6-month post–follow-up, no symptoms were reported, and clinical examination demonstrated that the edentulous ridge was completely healed. There was no sign of dehiscence. On cone-beam computerized tomography (CBCT), no adverse changes or ORN in radiological finding were seen ( Fig. 4 ).

## Discussion

Five-year survival rate for people with oral or oropharyngeal cancer is approximately 55% with many survivors living for decades without decreasing their ORN risk. 12 Analysis of epidemiological research on ORN does not offer precise data on the incidence and prevalence of ORN in the jaw due to a lack of agreement on its definition, differences in the length of follow-up between studies, and a lack of data from prospective studies. 5 The primary risk factors of ORN are the possibilities of “at risk” operations, such as tooth extraction and bone surgical insulation. Dental extractions have been identified as one of the most common beginning factors in the development of ORN in irradiated jaws. The rate of ORN after tooth extraction in irradiated patients is estimated to be between 2 and 18%. A radiation dose higher than 60 Gy and abuse of alcohol and tobacco are also known as etiological factors of ORN. 5 13 In addition, an increase in local inflammation and tissue infection are related to ORN as a consequence of dental caries and periodontal disease. 14

The pathophysiology of ORN is unclear and various hypotheses are used to explain ORN. Marx's initial suggestion of a nonhealing wound hypoxia, hypovascularity, and hypothesis of hypocellularity have lately been questioned, as were the findings of numerous other investigations not completely substantiated. 15 16 According to the RIF process, ORN is a radiation-generated fibroatrophic process, encompassing free-radical formation, endothelial dysfunction, inflammation, microvascular thrombosis, fibrosis and remodeling, and bone and tissue necrosis. 17 18 In various studies, the researchers used treatments based on the RIF hypothesis, and indicated that the condition is responsive to antioxidant treatment using combination of pentoxifylline and tocopherol. 17 18

Pentoxifylline, a methylxanthine derivative, has extensive use and vasodilatory benefits in patients with peripheral, vascular, and stroke conditions. Pentoxifylline has been proven to decrease inflammatory responses and platelet aggregation while increasing erythrocyte flexibility, hence lowering blood viscosity and coagulation potential. Vitamin E (tocopherol), which prevents the free radical production of lipid peroxidation, is a powerful antioxidant. Moreover, tocopherol is associated with fibrosis reduction. 19 As previously stated, novel therapy regimens have been proposed to reverse alterations in reactive oxygen species that causes RIF and eventually ORN.

Several reports have shown that the PRF can be used as an adjuvant to conventional nonsurgical periodontal treatment and as a regenerative material in oral implantation to elicit new bone formation. Tsai et al 20 reported that PRF can improve healing process in the patient with osteonecrosis of jaw, and it also promotes new bone formation. In addition, King et al 10 in 2019 reported early epithelization at 2-week postoperation in use of leukocyte-PRF (L-PRF) prophylaxis of ORN.

Further reports demonstrated its advantage, as it can express a range of activated signaling molecules, platelet-derived growth factor (PDGF), vascular endothelial growth factor (VEGF), and TGF-β. These growth factors are crucial for tissue regeneration and the tissue vascularization. 21 22 23

The biological effects of PRF in regenerative bone was investigated and proven, PRF can promote osteoblast attachment, proliferation through the Akt pathway, and matrix synthesis through the actions of heat shock protein 47. 24 Another concept for PRF (A-PRF+ or A-PRF + ), which is a new PRF-based matrix, which was described by Ghanaati et al. 9 A-PRF+ highly showed VEGF release when comparing with the A-PRF and conventional PRF after 7 days, and A-PRF+ also showed the highest concentration on day 10. 25

VEGF plays a key role in wound healing and tissue regeneration to encourage vascularization and the development of new vessels. 26 Sustained and improved VEGF release by A-PRF+ could therefore lead to greater regeneration and vascularization benefits, so providing a nutrition source to promote wound healing, and improve the pattern of bone and tissue regeneration. In addition, in 2017, Choukroun and Ghanaati 9 reported that using A-PRF+ increases the quantity of inflammatory cells and platelets and the growth factor in the matrices of the PRF.

Although the current case report resulted in satisfactory treatment outcome, there are several effective therapeutic strategies for improving healing process with combination of preoperative oral pentoxifylline/tocopherol and A-PRF+ wound closure. Further studies are needed to validate its therapeutic efficacy for radiated patients.

## Conclusion

Oral examination prior to radiation and regular dental check-ups following radiation can reduce the risk of ORN development. It is found that tooth extraction following radiotherapy greatly increases the risk of developing ORN. Tooth extractions following the radiation should be performed with minimal trauma, primary closure, and adjuvant therapy. Our case demonstrates that the combination of pentoxifylline/tocopherol and the A-PRF+ surgical approach is useful for would healing and prevention of ORN. Further studies are needed to validate its efficacy in preventing ORN in radiated patients.
